# The Ethnopharmacological Use of Mescaline for Psychiatric Disorders: A Systematic Review

**DOI:** 10.3390/ijms27073081

**Published:** 2026-03-28

**Authors:** Jonathan Shaw, Aidan Yong, Jacky Lee, Justin Cheng, Anton Andricioaei, Jen-Yeu Wang, Yaara Zisman-Ilani, Robert Bota

**Affiliations:** 1 School of Medicine, California University of Science and Medicine, Colton, CA 92324, USA; 2 Brain Health Solutions, Costa Mesa, CA 92626, USA; 3 Department of Anthropology, University of California, Riverside, CA 92521, USA; 4 Barnett College of Public Health, Temple University, Philadelphia, PA 19122, USA; yaara@temple.edu; 5 Lewis Katz School of Medicine, Temple University, Philadelphia, PA 19140, USA; 6 Division of Psychology & Language Sciences, University College London, London WC1H 0AP, UK; 7 College Medical Center, Long Beach, CA 2776, USA

**Keywords:** mescaline, Peyote, San Pedro cactus, psychedelics, psychiatry

## Abstract

Mescaline, the primary bioactive alkaloid found in Peyote and San Pedro cactus, has been used in traditional medicine for centuries and is now attracting renewed interest for clinical applications. The purpose of this systematic review was to search the literature for studies reporting the use of mescaline to address the gap in our understanding of mescaline use and its impact. References were exported from PubMed, Scopus, Embase, and Cochrane. Included studies contained patient data pertaining to mescaline, primary sources for beliefs on the use of mescaline as traditional medicine, and a range of psychiatric conditions. Excluded studies included unpublished studies, animal studies, and studies without English full-texts available. Of 2770 imported references, 66 met the inclusion criteria, with only 10 being found suitable for analysis. Studies reported therapeutic effects such as improvements in depression scales, well-being, nicotine dependence, alcohol use, and obsessions. Bayesian analysis found that certain effects were frequently reported, such as hypertension, headache, nausea, and vomiting. The existing literature on mescaline is limited and of highly variable quality, preventing definitive conclusions regarding the prevalence of psychological and somatic effects from mescaline and mescaline-containing ethnobotanicals. Additional research is needed to determine the safety profile of mescaline. Given the prevalence of Peyote use in the Native American Church, the collaboration of the Native American Church and regional hospitals/poison centers is recommended to create a registry to allow for standardized and clinically applicable data collection on the effects of mescaline in prevalent populations.

## 1. Introduction

Nonadherence to psychiatric medications remains a persistent challenge in mental healthcare, with rates ranging from 40 to 60% across various psychiatric conditions [1,2,3,4]. Among multiple contributing factors, medication side effects and perceived limited effectiveness drive many individuals to discontinue, reduce, or seek alternative treatments [5,6,7]. Mescaline (3,4,5-trimethoxyphenethylamine), a bioactive alkaloid found in Peyote (*Lophophora williamsii*) and San Pedro cactus (*Trichocereus pachanoi*), represents one such alternative that has been used in traditional medicine for centuries and is now attracting renewed clinical and research attention [8,9,10]. Mescaline is one of the oldest psychedelics studied by Western scientists, with Arthur Heffter first demonstrating that it is the primary active alkaloid in Peyote through a series of experiments in the late 1890s [11]. The Native American Church (NAC), with members stemming from tribes including the Navajo, has long been known to use Peyote not only as a sacrament in their religious practices, but also as a treatment for a variety of physical and psychological ailments [12,13,14]. With this context in mind, psychiatrists in the 1950s began to experiment with mescaline to study its potential psychotherapeutic effects for a variety of psychiatric disorders [11]. However, due in part to psychedelics’ association with the 1960s hippie counter-culture movement, as well as the increased regulations on clinical research that came with the Drug Amendments of 1962, psychedelic research in humans all but halted by 1976 and would not restart until 1994 [15]. This did not stop the consumption of mescaline and mescaline-related compounds by the general public, such as those in the rave or party scenes [16,17,18].

As psychedelic research expands for treatment-resistant depression, mescaline’s therapeutic mechanisms and potential risks require systematic evaluation, particularly given its distinct pharmacology and limited modern clinical investigation [15]. Like other psychedelics, mescaline primarily acts through the serotonin 5-HT_2A_ receptor as a partial agonist [19]. Mescaline also binds to the serotonin 5-HT_1A_ receptor, the α1-adrenergic receptor, the dopamine receptors D1/2/3, and the trace amine-associated receptor 1 [19]. Although the receptor activity of mescaline has been explored in both animal and human studies, a systematic review of modern clinical data regarding the use of mescaline for psychiatric purposes is missing [19].

The purpose of this systematic review was to search the literature for studies reporting the use of mescaline to address the gap in our understanding of mescaline use and impact, and to establish a baseline of the current literature as of 2026.

## 2. Methods

### 2.1. Inclusion and Exclusion Criteria

For this review, studies met the inclusion criteria if they included original patient data pertaining to mescaline/mescaline-containing plants, were primary sources for beliefs/opinions on the use of mescaline/mescaline-containing plants as traditional/indigenous medicine, and had English full-texts available. Additionally, in congruence with our search syntax, inclusion criteria consisted of a broad range of psychiatric conditions, including mood, anxiety, psychotic, and substance use disorders, along with relevant ethnopharmacological and traditional medicine contexts across all database searches. Exclusion criteria included unpublished studies (non-peer-reviewed studies or study protocols with no data), animal studies, and studies without English full-texts available. Only studies identified as quantitative or mixed methods were included in this review.

### 2.2. Search Strategy and Study Eligibility

The protocol of this review was prospectively registered in PROSPERO as CRD420251184986. The full search syntax databases, including keywords and MeSH terms, can be seen in Supplementary File S1. Using the Covidence systematic review management program, two researchers independently evaluated each reference against the inclusion/exclusion criteria, with any disagreements being adjudicated by a third researcher. A completed PRISMA checklist can be seen in Supplementary File S4 [20].

### 2.3. Data Extraction and Quality/Risk of Bias Assessment

Two researchers independently conducted data extractions, which were compared in order to form a consensus. Any disagreements between the data extractions were adjudicated by a third researcher. Similarly, quality and risk of bias were assessed by two researchers, with disagreements being adjudicated by a third researcher using the Joanna Briggs Institute’s Checklist for Case Reports, the National Institute of Health’s study quality assessment tools (Observational Cohort and Cross-Sectional Studies; Case Series Studies), and the Risk Of Bias In Non-randomized Studies—of Interventions [21,22,23].

### 2.4. Analysis

Heterogeneity in studies was assessed using the Higgins Index (I^2^). Due to the significant variance in historical reporting and sample sizes, a Bayesian framework was utilized to provide a more robust interpretation than simple frequency reporting. This approach allows for the integration of high-fidelity clinical trials as ‘priors’, ensuring that small sample case reports do not disproportionately skew the safety profile. A Gaussian–Poisson prior was also created based on established clinical baselines to operationalize ‘frequently reported’ effects as those exceeding a 95% credible interval. This quantifies the conditional probability of adverse events, demonstrating that risk is a function of the clinical environment rather than an inherent property of the substance. All analyses and subsequent visualizations were performed in Python 3.10 using Pandas, NumPy, SciPy, and Matplotlib as libraries.

## 3. Results

**Figure 1:**
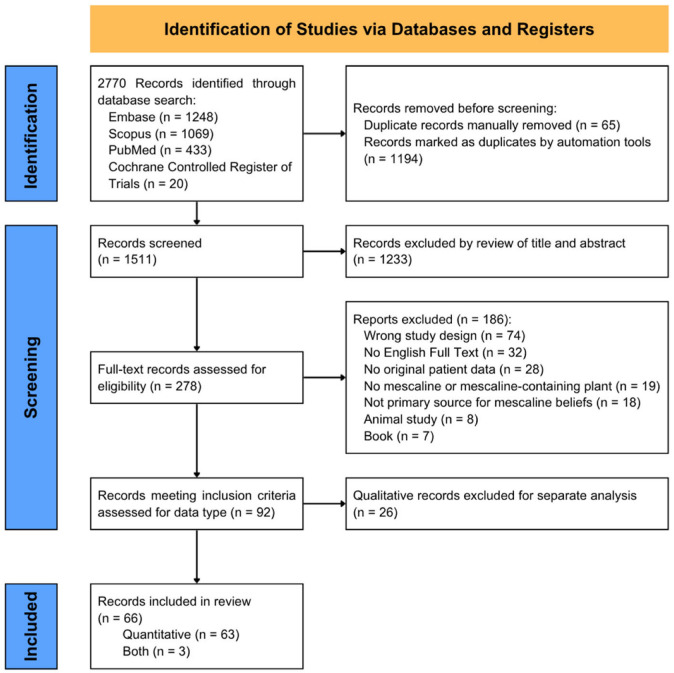
PRISMA flowchart.

Using the search terms detailed in the registered protocol, 2770 studies were imported for screening from Embase (1248), Scopus (1069), PubMed (433), and the Cochrane Controlled Register of Trials (20) on 27 October 2025. Of the 66 references [12,13,16,17,18,24,25,26,27,28,29,30,31,32,33,34,35,36,37,38,39,40,41,42,43,44,45,46,47,48,49,50,51,52,53,54,55,56,57,58,59,60,61,62,63,64,65,66,67,68,69,70,71,72,73,74,75,76,77,78,79,80,81,82,83,84] that met inclusion criteria for this quantitative review, 9 (13.6%) were case reports, 4 (6.1%) were case series, 34 (51.5%) were observational/cross-sectional cohort studies, and 19 (28.8%) were clinical trials. Details regarding reference selection and reasons for exclusion are presented in Figure 1, while the demographic information and findings of the included studies are presented in Table 1. Further details relating to the included references can be seen in Supplementary File S2.

### 3.1. Quantitative Analysis

Of the ten studies that were included in the final Bayesian Analysis, there was one controlled clinical trial, two clinical observations, one case series, one clinical pilot, one retrospective case series, one individual case report, and one modern Phase I study. The analysis was then split into two parts, one for psychological effects and another for somatic effects. Both groups were further split into categories based on whether the data was clinical (Group 1) or through exposure (Group 2) as well.

This stratification was chosen to account for the inherent reporting bias found in historical data, where unsupervised exposures are typically only documented when they result in medical crises or poison center calls. By separating these from controlled clinical observations, the analysis can distinguish between the core effects of mescaline and the adverse outcomes associated with high-stress, unsupervised environments. This grouping allows for a more accurate assessment of how the context of use influences the frequency and severity of reported symptoms.

**Figure 2:**
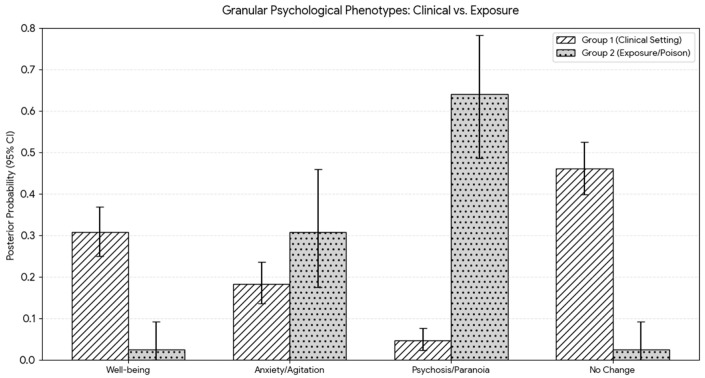
Granular psychological phenotypes. Comparison of modeled psychological outcomes between supervised medical settings and unsupervised exposures. The *Y*-axis represents the Posterior Probability (0.0 to 1.0), reflecting the calculated likelihood of a psychological state manifesting based on the weighted integration of study reliability and sample size. Bar heights indicate the Mean Posterior Probability for each phenotype, representing the most likely occurrence rate within that setting. The vertical whiskers denote the 95% Credible Interval (CrI), identifying the range in which the true probability resides with 95% certainty. Narrower intervals indicate high consensus across the literature, while wider intervals (e.g., psychosis in Group 2) reflect greater uncertainty due to smaller sample sizes or conflicting case reports.

From the eight studies with data on psychological effects (Group 1: N = 215, Group 2: N = 33), the Bayesian Analysis returned the following results, showcased in Figure 2 [31,32,33,34,52,59,69,77,81]:

These results show that the majority of the time, mescaline produces negative effects as a result of exposure instead of being administered clinically. Interestingly, in clinical settings, it likely creates a sense of well-being, as seen in Table 2 [32,33,34,52,69]. These differing results between the two groups show that the environment in which mescaline is administered contributes greatly to the results. However, the data regarding exposure are likely subject to reporting bias, as anomalous negative outcomes are more likely to be documented.

**Figure 3:**
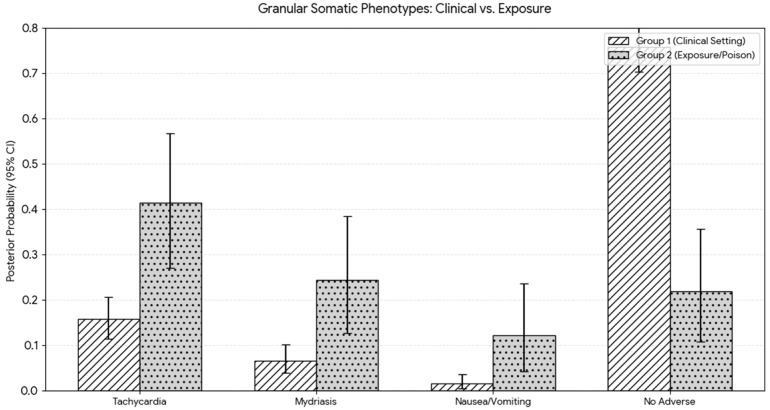
Granular somatic phenotypes. Comparison of modeled physiological outcomes between supervised medical settings and unsupervised exposures. The *Y*-axis displays the Posterior Probability (0.0 to 1.0), providing a weighted estimate of the likelihood of a somatic symptom occurring while adjusting for the precision of the source data. The height of each bar represents the Mean Posterior Probability for a given somatic marker within that specific environment. The vertical whiskers represent the 95% Credible Interval (CrI), illustrating the statistical uncertainty of the estimate. Tight intervals (e.g., cardio) signify consistent reporting across the dataset, whereas wider intervals highlight phenotypes with higher variance or limited granular data in the available literature.

From the eight studies that detailed somatic effects (Group 1: N = 181, Group 2: N = 8), the Bayesian Analysis gave the following results, showcased in Figure 3 [31,32,33,34,52,56,59,77,81]:

The difference between environments is even more apparent when it comes to the somatic effects, as seen in Table 3. There is significant overlap when it comes to having no adverse reactions to mescaline in [32,33,34,59,82]. Additionally, some of the physical effects, such as an increase in heart rate, are predictable and therefore can be addressed [56]. It is also important to note that the increase in probability pertaining to nausea and vomiting is likely due to the different-sized populations, as three cases were reported in the clinical group compared with four in the smaller exposure group. Overall, mescaline has a high physical safety margin, especially regarding clinical administration of the psychedelic.

#### Reasons for Exclusion from Analysis

Most case reports discuss potential side effects associated with mescaline, suggesting the need for caution for its use/administration [30,31,39,41,72,77,81]. Many additional studies examine psychedelics as a class in a broader sense, with mescaline as an undifferentiated part of their analyses [16,17,24,44,68,75,80,84]. There is also a notable number of studies that come from coarse data, which makes them ineligible for inclusion due to the self-reported nature and non-generalizability of the data [16,17,24,44,45,46,47,48,49,50,51,68,75,80,83].

### 3.2. Bias and Quality Assessment

The bias and quality assessments for each included reference are presented in Supplementary File S3, with a summary provided below. Of the nine case reports, four (44.4%) were of good quality, one (11.1%) was of moderate quality, and four (44.4%) were of poor quality. Among the four case series, two (50%) were of good quality, one (25%) was of moderate quality, and one (25%) was of poor quality. Of the 34 observational/cross-sectional cohort studies, 16 (47.1%) were of good quality, and 18 (52.9%) were of moderate quality. Of the 19 clinical trials, 15 (78.9%) were identified as having a serious risk of bias in reporting, 2 (10.5%) were at moderate risk, and 2 (10.5%) were at low risk of bias.

## 4. Discussion

### 4.1. Current State of Evidence

As of 2026, the current state of the literature on mescaline and mescaline-containing ethnobotanicals, such as Peyote, suggests potential therapeutic relevance; however, it remains insufficient for formal clinical decision making. Studies examined in this review have reported therapeutic effects associated with mescaline that have included improvements in depression scales, well-being, nicotine dependence, alcohol use, and obsessions [12,36,39,47,57]. On the other hand, studies have also consistently reported physical effects such as hypertension, headache, aggression, nausea, and vomiting [31,34,35,37,41,52,58,59,70,82]. However, some of these physical effects, such as nausea, hypertension, and headache, are also commonly reported with other classic psychedelics like Lysergic acid diethylamide (LSD) and psilocybin, potentially indicating that these physical effects are not unique to mescaline [52].

Both the therapeutic and physical effects should be interpreted cautiously, as the claims remain largely speculative without well-controlled large clinical trials. Despite the relative consistency of these reported effects, the extent to which the authors can interpret the results meaningfully is constrained due to substantial limitations in the current body of literature. These limitations include various factors such as publishing year, study design, and sample size.

Additionally, as noted in Table 2, the setting in which mescaline is administered appears to influence its reported effects. Mescaline administered in clinical settings is more likely to produce a sense of well-being [32,33,34,52,69]. This observation suggests that the expectations of individuals consuming mescaline or mescaline-containing ethnobotanicals, such as Peyote or San Pedro cactus, may influence their overall experiences and perceived effects. However, due to the limited sample sizes available for analysis, the effects of mescaline, Peyote, and San Pedro cactus could not be differentiated in this review. Therefore, when interpreting the current literature and considering future research on mescaline and related ethnobotanicals, careful attention should be given to the specific substance used as well as the cultural and clinical contexts in which it is administered.

For example, the use of pure mescaline, whether isolated or synthesized, differs from the use of ethnobotanicals such as Peyote or San Pedro cactus, because these plants vary in their mescaline content and contain additional bioactive compounds [85]. Furthermore, perceptions of “negative” versus “positive” effects associated with psychedelic use may vary depending on the cultural background and setting of the user. For instance, ceremonial Peyote users from rural northcentral Alabama have reported that part of the value of the Peyote ceremony comes from successfully enduring the mental and physical challenges associated with its use [10]. Despite this, the current clinical literature has not extensively considered two important factors: the differences between pure mescaline and mescaline-containing ethnobotanicals, and the cultural or clinical settings in which these substances are used. These considerations are particularly important for clinicians when discussing mescaline, Peyote, or San Pedro cactus use with patients, as the available safety data may not reflect the intentions or contexts in which patients are actually using these psychedelics.

Additionally, clinicians should also take into account that analysis of data from the existing literature is significantly limited, particularly due to the decades-long halt in psychedelic research. The majority of available clinical trials were conducted 50 years ago, while more modern studies are either set in non-clinical settings (correlational survey studies) or lack adequate sample sizes (case reports and case series) to allow for conclusive results. As a result, many studies that initially met inclusion criteria were excluded from quantitative analysis due to multiple reasons, such as grouping results of mescaline with other psychedelics [16,17,24,44,68,75,80], general use of mescaline as a recreational drug or for religious reasons [10,13,25,29,30,37,38,55,60,64,72,74,77,86], or use of mescaline to induce psychological symptoms [39,41,42,63,66,73].

Of particular note, several correlational survey studies examined the use of mescaline or Peyote and their association with various psychiatric disorders [47,48,49,50,83]. These studies were conducted using the National Survey on Drug Use and Health, but used identical methodology for analysis. This provided additional difficulties in including this research into the analysis, as it provided multiple examples using the same data with small differences in outcome. While each paper focuses on different specifics, the data is inherently coarse and also inflates the potential pool of data used for this synthesis. On the other hand, correlational survey studies are also susceptible to bias arising from participant self-selection and social desirability effects [87,88]. Psychedelic use has been shown to increase the likelihood that prospective participants will volunteer for studies examining psychedelic use, including single unpaid surveys, multiple unpaid surveys presented longitudinally, or unpaid administration studies. In contrast, other demographic variables do not reliably predict consistent participation in future psychedelic research [87]. Additionally, survey participants who report lower severity of psychiatric symptoms or disorders, such as depressive symptoms or alcohol use, also tend to report greater concern about social desirability when completing surveys [88]. These factors raised concerns among the authors regarding the generalizability of findings that associate psychedelic use with reduced odds of experiencing a non-severe major depressive episode within the past year [50]. Furthermore, many of these correlational studies focused on lifetime use of individual psychedelics but did not report analyses examining how the frequency of psychedelic use or polysubstance exposure relates to psychiatric conditions. They also did not establish temporal relationships between psychedelic use and the psychiatric outcomes measured [47,48,49,50,83].

As a result of these limitations, this review’s analysis only consisted of approximately 300 individuals between both psychological and somatic effects analysis, a notably low sample size. This significantly limits the generalizability of reported findings and constrains the interpretation of the results. Beyond these issues regarding scope and scale, additional concerns related to variability in study quality assessment and risk of bias further limit the rigorous evaluation of the existing mescaline literature.

### 4.2. Risk of Bias, Quality Concerns, and Class-Level Comparison

In addition to the aforementioned structural limitations, there was also significant variation regarding the quality and risk of bias of the included studies. The majority of the clinical trial data originated from studies from the 1950s, many with serious overall risk of bias related to selection of participants, measurement of outcomes, or selection of reported results [32,33,34,35,36,40,56,59,69,73,82]. A sizable proportion of the case reports and case series were also noted to be of overall poor quality rating due to various reasons, including lacking clear descriptions of patient history, assessment methods, and interventions [12,27,62,77,79].

Concerns regarding study quality are further heightened by population selection, as many reported benefits of mescaline use were derived from populations without formal diagnoses, limiting conclusions regarding the therapeutic efficacy of mescaline [45,46,47,48,49,50,51,83]. Additionally, several newer cohort studies relied on survey data that were not clinically applicable [45,47,48,49,50,51,83]. This methodology by researchers, who have published extensively examining how survey data correlate with psychiatric disorders, complicates future meta-analyses, given their lack of clinical applicability and findings that lack definitive causality.

On the other hand, more recent clinical trials are high quality in terms of methodology and reporting, though they are lacking in terms of sample size [52,58]. These limitations, for much of the existing literature, substantially limit the authors’ analysis of the existing data on mescaline/Peyote/San Pedro cactus. This further emphasizes the need for large-scale clinical trials to investigate the psychological as well as physical effects of mescaline/mescaline-containing ethnobotanicals in a replicable and high-quality manner. Collectively, these concerns regarding severe risk of bias and poor quality obfuscate whether the reported psychological and physical effects from the literature are uniquely attributable to mescaline or are pharmacodynamic properties shared among classic psychedelics. Importantly, this further stresses the importance of the systematic evaluation of risk of bias and quality to limit the overestimation of therapeutic benefits and to reliably interpret effects specific to mescaline compared to those across all classic psychedelics.

### 4.3. Mescaline in the Context of Other Classic Psychedelics

Mescaline belongs to the phenethylamine class of psychedelics, whereas LSD and psilocybin are tryptamines, containing an indole ring derived from tryptophan and structurally similar to serotonin [89,90]. This fundamental difference in chemical scaffold is important because tryptamines closely mimic serotonin’s indole pharmacophore, while phenethylamines, such as mescaline, interact with serotonergic receptors through a different structural framework [89].

These structural differences are associated with substantial differences in pharmacokinetics and duration of action, as demonstrated through experimental comparisons in humans, which found that while mescaline, LSD, and psilocybin produce broadly similar psychedelic phenomenology, they differ in onset, duration, intensity profiles, and autonomic effects [89,91]. Mescaline produces effects that can persist for about 11.1 h while LSD lasts 8.2 h and psilocybin lasts 4.9 h [52,92,93,94]. Such differences reflect variations in metabolism, lipophilicity, receptor affinity, and required dosing, which justifies further research into mescaline individually, especially since it remains highly prevalent in certain populations, such as members of the NAC [94].

Taken together, mescaline’s phenethylamine structure, substantially longer duration of action, and distinct receptor affinity and pharmacodynamic profile indicate that it cannot be fully represented by findings derived from tryptamines such as psilocybin [94]. Although grouped within the broader category of serotonergic or “classic” psychedelics due to shared 5-HT_2A_-mediated effects, these differences support the need for independent investigation of mescaline rather than subsuming it under generalized psychedelic mechanisms [89,92,94].

### 4.4. Future Directions and Research Implications

Future studies examining mescaline or mescaline-containing ethnobotanicals should build upon the small, but high-quality clinical trials identified by this review to provide more standardized, comparative, and ethically based research [52,58]. Although mescaline remains restricted for medical use, the long-standing ceremonial and cultural use of Peyote by the NAC further emphasizes the need for continued research into mescaline/Peyote as its use remains prevalent in these populations/cultures. However, before therapeutic investigations can occur, the safety profile of mescaline must be further investigated in large, well-controlled clinical trials.

A recent Phase I study of mescaline has demonstrated that a single dose of mescaline to healthy individuals is safe regarding psychological and physical harm in a controlled clinical setting, but this finding is limited due to a relatively small sample size (N = 48) [52]. Future efforts should build upon the existing Phase I studies identified by this review to fully establish a comprehensive safety profile by documenting pharmacokinetic and dynamic properties and defining dose-dependent effects.

Furthermore, comparative studies directly evaluating mescaline versus other classic psychedelics, including LSD and psilocybin, are recommended and essential to distinguish compound-specific effects from class-general classic psychedelic responses. Lastly, registries in collaboration with medical centers and poison control networks serving areas with high prevalence of Peyote use, with a partnership with the NAC, would be highly beneficial in being able to provide real-world safety profiles, further advancing the research into mescaline while maintaining ethical and cultural boundaries. These registries could collect longitudinal clinical data through the primary care physicians and affiliated hospital systems of NAC members who voluntarily choose to participate. By providing anonymized, clinician-sourced information on the psychological and physical health of members who regularly participate in Peyote ceremonies, the registry would enable more granular and reliable data collection. This information could be standardized through validated clinical measures such as the Patient Health Questionnaire-9 and the Generalized Anxiety Disorder-7. In addition to psychological measures, objective clinical metrics such as laboratory values could also be tracked and compared with those of non-Peyote users. Although concerns about self-selection bias may remain—as not all NAC members may feel comfortable participating in such registries—the American Indian Religious Freedom Act Amendments of 1994 legally protect the use of Peyote within the NAC. This legal protection may help reduce social desirability bias among participants when reporting their experiences and health outcomes.

In order to integrate mescaline and mescaline-containing ethnobotanicals into modern psychiatry practice, future studies should aim to standardize clinical research of mescaline and hence bridge the gap between psychedelic exceptionalism and other psychopharmacologic agents. Altogether, these findings support the need for continued rigorous investigation to establish mescaline’s safety, efficacy, and overall compound-specific profile within the broader class of classic psychedelics.

#### Limitations

Despite the various strengths of this review, certain limitations warrant consideration. First, only including publications in English may have omitted relevant references from Latin America and other non-English speaking regions where mescaline use is common and has cultural significance. Second, a majority of the clinical trials collected were outdated due to the hiatus in psychedelic research and may have a serious risk of bias in their reporting. Furthermore, a majority of studies consisted of observation or cross-sectional cohort studies, which limit the ability to establish causality. Lastly, heterogeneity in how mescaline and Peyote as individual substances were reported and analyzed in some references limits the generalizability to classic psychedelics as a category.

## 5. Conclusions

The current landscape of mescaline literature is marked with significant quality limitations and risk of bias concerns, preventing this review from formulating concrete statements regarding the therapeutic effects and potential adverse effects of mescaline or mescaline-containing ethnobotanicals. Although psychological (improvement of depression symptoms, enhanced sense of well-being, anxiety, etc.) and somatic (diaphoresis, hypertension, nausea, and vomiting) effects are frequently reported in the literature, an accurate prevalence of these effects cannot be determined until further research is conducted. Large, high-quality clinical trials are needed to better define the safety profile of mescaline and its associated ethnobotanicals (Peyote and San Pedro cactus) before therapeutic studies can be conducted. Of note, the context and setting of mescaline use appear to meaningfully influence its psychological and physical effects, warranting further systematic evaluation. The clinical setting, cultural context, and the specific substance consumed, whether pure mescaline or an ethnobotanical source such as Peyote or San Pedro cactus, represent variables that the existing literature has largely not considered. One way to address this gap would be to establish a standardized registry of regular Peyote and San Pedro cactus users, particularly in areas where the Native American Church is present. Such a registry could collect clinician-sourced longitudinal data within the cultural and religious contexts in which Peyote and San Pedro cactus are regularly consumed. This approach would generate clinically applicable data that could quantify the frequency of Peyote and San Pedro cactus consumption and clarify the temporal relationship between psychedelic use and associated side effects. Researchers have identified the absence of such data as a key limitation of prior correlational, cross-sectional survey studies. Ultimately, continued development of high-quality research will be essential for evaluating mescaline and advancing the field of psychiatric care.

## Figures and Tables

**Table 1:** Summary of Included References.

Reference	Study Type	Bias Rating Scale/ Overall Score	Notes
Acevedo 2025 [24]	Observational-cohort study	NHLBI/Moderate	Results of 270 reports did not differ between phenethylamines (mescaline) and tryptamines. Psychedelics help people gain psychological insight with no adverse effects noted.
Agin-Liebes 2021 [25]	Cross-sectional survey study	NHLBI/Moderate	Cross-sectional survey of 452 participants found improvements in psychiatric conditions, including depression, anxiety, PTSD, and alcohol/drug use disorders associated with insight, ego dissolution, and mystical-type experiences. “Low rates of adverse experiences”.
Albaugh 1974 [12]	Case Report	JBI Case Report/Poor	Predominantly discusses the Native American Church and the potential for psychodynamic effectiveness of Peyote. Very brief and sparsely detailed case on the use of Peyote for cessation of alcohol use disorder.
Argento 2022 [26]	Longitudinal, prospective cohort	NHLBI/Moderate	Report of 229 psychedelic users for opioid use disorder; only 2 participants used mescaline. Unable to differentiate between the effects of mescaline and those of other classic psychedelics.
Bergman 1971 [13]	Case series, ethnography	NHLBI/Moderate	Estimated incidence of serious adverse reactions to Peyote was 1 in 70,000 users. In total, 200 cases were observed, and 5 reported cases of adverse reactions. Case 1: Male (mid-20s) drank alcohol, then attended a Peyote meeting where he experienced paranoid delusions that others wanted to kill him. He ran away and broke into his house, injuring himself, despite having a key. Was treated with IM chlorpromazine and all symptoms of psychosis/anxiety resolved within 24 h. Case 2: Male (early 20s) developed an acute schizophrenic episode about the time of a meeting; he had been to many previous meetings without incident. Was markedly successful with inpatient treatment and has attended many more meetings without difficulty. Case 3: 22-year-old male complained of free-floating, anxiety, and depersonalization associated with Peyote meetings. After stopping the meetings, his symptoms decreased. Cases 4 and 5: Chronic schizophrenia patients who became acutely anxious during meetings, but many other chronic schizophrenics attend meetings without adverse effects.
Blum 1977 [27]	Case series	NHLBI/Poor	Predominantly discusses the alkaloid mechanisms for Peyote use and briefly discusses 3 cases where the use of Peyote in the Native American Church was able to improve the lives of those with opioid use disorder.
Boehnke 2023 [28]	Cross-sectional survey study	NHLBI/Moderate	A total of 1221 surveys revealed that patients believe psychedelics help manage mental health, with many coinciding with psych medicine use; desire for professional support (62% depression, 52% anxiety, 27% ADHD). Of the sample, 54% of respondents who combined psychedelics and psychiatric medications report a risk.
Bohn 2023 [29]	Cross-sectional survey study	NHLBI/Moderate	A total of 42 surveys conducted at San Pedro cactus ceremonies in the Netherlands produced spiritual experiences, moderate ego dissolution, and ± consciousness states measured by the altered states of consciousness rating scale, the ego-dissolution inventory, the challenging experience questionnaire, and the mystical experience questionnaire. Adverse effects of physical distress, impaired control and cognition are reported.
Borkel 2024 [30]	Cross-sectional survey study	NHLBI/Moderate	A total of 1022 surveys from Spanish-speaking participants found that psychedelics, including mescaline, for therapeutic or spiritual purposes, were associated with less anxiety, depression, greater wellbeing, and mindful acceptance in spiritual/religious use, while escapist use had the inverse.
Carstairs 2010 [31]	Retrospective cohort	NHLBI/Moderate	Retrospective chart review of a poison center examining 31 cases of single-substance ingestion of Peyote/mescaline, reported only adverse effects of hallucinations, tachycardia, agitation, mydriasis, hypertension, nausea, paranoia, psychosis, vomiting, and generalized seizure. Did not include reasons for ingestion. Treatments for mescaline intoxication included IV fluids, benzodiazepines, and antipsychotics (droperidol). All symptoms resolved within 24 h except for 1 patient who had persistent tachycardia, hypertension, and agitation that lasted 72 h.
Cattell 1954 [32]	Single-arm trial	ROBINS-I/Serious	A total of 59 participants, split into 3 groups. Group 1: Pseudoneurotic schizophrenia group was more productive psychodynamically under the influence of mescaline. Group 2: The frankly schizophrenic group saw limited benefits psychodynamically, with only 8/26 shedding light on their illness and personality structure. Group 3: The deteriorated schizophrenia group saw only 5/16 providing some pertinent psychodynamic material under the influence of mescaline.
Denber 1956 [33]	Within-subjects study	ROBINS-I/Serious	A total of 42 participants were given IV mescaline 0.5 g with or without phenothiazine pre-medication: chlorpromazine, diethazine, promethazine, or saline. Anxiety was necessary in the therapeutic process. Overall, 8 patients improved, 1/8 chlorpromazine, 1/4 diethazine, and 1/2 saline before mescaline.
Denber 1955 [34]	Single-arm trial	ROBINS-I/Serious	A total of 25 Schizophrenic inpatients received 0.5 mg IV mescaline sulfate w/EEG before and after ECT. One reported case of complete remission of schizophrenic symptoms and three cases with improvement of psychotic symptoms. Adverse effects of anxiety, hostility, negativism, psychosis, nausea, vomiting, sweating, and catatonia were reported.
Denber 1955 [82]	Parallel-group randomized controlled trial	ROBINS-I/Serious	A total of 57 participants in RCT were subdivided into chronic and acute groups. Adverse effects reported were anxiety, tension, delusions, hallucinations, increases in BP, pupil dilation, nausea, and SI. Conditions treated included schizophrenia, psychoneurosis, involuntary psychosis, manic depressive disorder, and psychosis.
Denber 1954 [35]	Within-subjects study	ROBINS-I/Serious	A total of 10 subjects were given 0.5 g of mescaline intravenously and questioned. After one to one and a half hours of observation, subjects were given 50 mg of chlorpromazine hydrochloride IM. They were then interviewed at the second and 24th hour following the chlorpromazine injection. Afterwards, they were seen on daily rounds and given psychotherapeutic sessions by residents. Out of 10 participants, 7 had marked conflict or confusion in the “sexual sphere” as evidenced verbally or by gesture. The chlorpromazine diminished or resolved the anxiety/tension caused by mescaline within an hour after injection for 8/10 participants. One patient developed an acute rage reaction that necessitated physical restraints for her.
Denber 1958 [36]	Single-arm trial	ROBINS-I/Serious	Nine participants were given 0.5 g of mescaline sulfate injections, and eight of them were interviewed the same day. The last participant was so disturbed by the mescaline that he was only interviewed 7 days after the injection. Hysteria and depression scales showed a lessening of scores after taking mescaline compared to baseline. However, it was noted that those taking mescaline become more paranoid, anxious, and show psychopathic/schizophrenic trends while under its effects.
Desai 2022 [16]	Cross-sectional survey study	NHLBI/Good	All hallucinogens, including mescaline, were collated into binary hallucinogen use vs. no hallucinogen use groups. Adolescents with hallucinogen use histories were found to have a higher prevalence of feeling hopeless (48.4 vs. 27.8%), considering suicide (36.3% vs. 15.1%), and attempting suicide that required medical attention (12.0 vs. 1.5%) (*p* < 0.0001 for all). Hallucinogen users had a higher prevalence of other substance use.
Duraković 2022 [37]	Case report	JBI Case Report/Good	A 41-year-old male searching for the meaning of life consumed mescaline tea and thick cactus paste during a “shamanic session” and entered a state of catatonia approximately 50 h ingestion. Received risperidone PO 2 mg. Then, aripiprazole and clozapine with diazepam were tried, which resulted in a gradual improvement of memory of events leading to presentation to the emergency department. Was discharged on aripiprazole 10 mg BID, clozapine 25 mg TID, and diazepam 5 mg PR QHS. Outpatient follow-up gradually omitted psychopharmacology without any further symptoms or side effects.
Halpern 2005 [38]	Cross-sectional observational study	NHLBI/Good	A total of 176 subjects were subdivided into Peyote, former alcoholic, and comparison groups. The former alcoholic group showed greater deficits in the Rey–Osterreith Complex Figure Test compared to the Peyote group (SE was −4.0 [1.3]; *p* = 0.002). No associations between log-transformed lifetime Peyote use and any neuropsychological test measure approached significance. However, greater lifetime Peyote use was associated with significantly better scores on 5 of 9 scales on the Rand Mental Health Inventory.
Hermle 1992 [39]	Non-blinded clinical trial	ROBINS-I/Moderate	A total of 24 participants (12 mescaline) reported that their Brief Psychiatric Rating Scale total score changed significantly during the experiment (tot3; measured by Friedman rank ANOVA; df = 3; X^2^ = 20.30, *p* < 0.001). The increase was most prominent in the subscales thought disorder (THOT; *p*—0.0001) and activation (ACTV; *p* = 0.004). Furthermore, significant changes were found in the subscales of anergia (ANER; *p* = 0.034) and anxiety/depression (ANDP; *p* = 0.023). The self-rating score “Delusions” (Paranoid Depression Scale) was also significantly changed (Friedman rank ANOVA; df = 3; X^2^~−8.82~*p*~0.032).
Hoch 1952 [40]	Single-arm trial	ROBINS-I/Serious	A total of 59 participants, mainly descriptive reports regarding the effects of mescaline on the comorbid conditions, including obsessions, anxiety, and depression, of multiple patients with schizophrenia. Reports demonstrated that obsessions improved with an increased willingness to discuss experiences. Adverse effects of hallucinations, hostility, and paranoia.
Hollister 1962 [41]	Multi-arm, multi-stage trial	ROBINS-I/Serious	A total of 20 participants were given either mescaline 5 mg/kg, LSD-25 1 mcg/kg, or psilocybin 150 mcg/kg. Only 18 participants received all 3 drugs. Mescaline evoked the most somatic effects compared to LSD and psilocybin; 18 with dizziness, 14 with weakness/fatigue, 13 with hot/cold feelings, 15 with decreased appetite, 14 shaking, 18 nausea, 9 drowsiness, 13 paresthesias, 11 heaviness/lightness of extremities, 15 ill feeling/malaise, 6 blurred vision, 10 change in sweating, and 11 decreased salivation.
Hollister 1964 [42]	Factorial trial	ROBINS-I/Serious	A total of 24 participants were given LSD 1.5 mcg/kg, mescaline 5 mg/kg, and psilocybin 225 mcg/kg either by themselves or as a combination. Compared to LSD and psilocybin, mescaline had more of a tendency to decrease scores for friendliness, energy, and clear-thinking during the first 2 h after ingestion when comparing pre-drug scores. During the fourth hour, these scores would increase to more than pre-drug scores. Additionally, mescaline was slightly more likely to cause psychosomatic symptoms such as jitteriness. The study used the Clyde mood scale to determine mood factors.
Hutten 2019 [43]	Cross-sectional survey study	NHLBI/Moderate	A total of 1116 participants (887 currently microdosing, 229 history of microdosing, 187 history of mescaline use); 20.2% reported negative effects while microdosing. No statistically significant difference between current and past users with respect to psychological effects. All psychedelics were analyzed as a group, so there are no separate mescaline results.
Johansen 2015 [44]	Cross-sectional survey study	NHLBI/Moderate	Cross-sectional survey study of 135,095 participants examining the association between classic psychedelics and mental health, no association between lifetime psychedelic use and mental health problems/suicidal behaviors was identified.
Jones 2023 [45]	Cross-sectional survey study	NHLBI/Good	Cross-sectional survey study of 5252 participants found that mescaline, but not Peyote, was noted to increase the odds of engaging in fewer important activities, which is one of the signs of hallucinogen dependence.
Jones 2022 [46]	Cross-sectional survey study	NHLBI/Good	Cross-sectional survey study of 262,617 adolescents found that mescaline was not associated with lifetime suicidal thinking, planning, or attempts.
Jones 2022 [47]	Cross-sectional survey study	NHLBI/Good	Cross-sectional survey study of 214,505 adults found that mescaline was broadly associated with decreased nicotine dependence across all domains, while Peyote was associated with more limited decreased nicotine dependence across two domains.
Jones 2023 [48]	Cross-sectional survey study	NHLBI/Good	Cross-sectional survey study of 214,505 adults found that mescaline, but not Peyote, was found to be associated with lower odds of having difficulties dealing with strangers.
Jones 2022 [49]	Cross-sectional survey study	NHLBI/Good	Cross-sectional survey of 214,505 adults found that only Peyote was associated with a lowered odds of cocaine use disorder; lifetime mescaline use did not have a significant odds ratio.
Jones 2022 [50]	Cross-sectional survey study	NHLBI/Good	Cross-sectional survey study examining the association with classic psychedelic use and major depression. Mescaline and Peyote were not associated with significant odds of major depressive episodes within the past year, nor lifetime episodes.
Jones 2022 [51]	Cross-sectional survey study	NHLBI/Good	Cross-sectional survey study examining the association between classic psychedelic use and crimes. Mescaline was associated with lower odds of possession/selling drugs, while Peyote was associated with lower odds of vehicular theft or driving under the influence.
Jones 2022 [83]	Cross-sectional survey study	NHLBI/Good	Cross-sectional survey of 214,505 adults examining the association between classic psychedelics and opioid use disorder. Mescaline and Peyote were not associated with increased or decreased odds of opioid use disorder.
Karp Barnir 2025 [17]	Cross-sectional survey study	NHLBI/Moderate	Survey of 343 participants. Mind-altering substances were grouped together as classic psychedelics vs. MDMA vs. no substance use; therefore, it was difficult to derive mescaline-specific results. Also, some participants took multiple substances, but the data did not specify which participants had multiple substance use, rather just a checklist of whether they did or did not use psychedelics.
Klaiber 2024 [52]	Double-blind, placebo-controlled, crossover study	ROBINS-I/Low	This reference comprises two clinical trials (NCT04227756 and NCT04849013) that examine mescaline. Study 1 compared the acute effects of LSD, psilocybin, and mescaline in 32 health participants, with each participant receiving a single dose of each substance (plus a placebo) in counterbalanced order. Study 2 examined the subjective effects of different doses of mescaline (100, 200, 400, and 800 mg) and the role of the 5-HT2A receptors in mescaline-induced altered states of consciousness using the 5-HT2A antagonist ketanserin. In Study 2, 16 participants each received 100–800 mg mescaline and a placebo. They additionally received 800 mg of mescaline with ketanserin. The studies used visual analog scales to report subjective changes and the 5 Dimensions of Altered States of Consciousness. Adverse effects were reported using the List of Complaints that had previously been used in other clinical trials.
Korman 2023 [53]	Cross-sectional survey study	NHLBI/Good	Cross-sectional survey study of 199,405 adults examining the association between classical psychedelic use and motivation-based work-absenteeism. Before accounting for covariates, lifetime classic psychedelic users reported higher rates of motivation-based workplace absenteeism. However, mescaline/Peyote/San Pedro cactus use was not associated with motivation-based workplace absenteeism.
Krebs 2013 [54]	Cross-sectional survey study	NHLBI/Good	Cross-sectional study of 130,152 adults examining the use of classic psychedelics and their association with serious psychological distress/mental health. K6 scale for quantifying psychological stress. Lifetime mescaline use is associated with lower rates of serious psychological distress and lower psychiatric medication prescriptions. Mescaline/Peyote was also associated with lower rates of symptoms of agoraphobia.
Lu 2004 [55]	Case report	JBI Case Report/Good	Case report of a 54-year-old male with no prior history of psychosis presented with a 2-week history of psychotic symptoms that began within hours of drinking Peyote juice during a healing ceremony. The patient has a history of alcohol abuse and combat-related PTSD, both in remission for 20 years. The urine drug screen was negative. Symptoms resolved upon waking up after 50 mg of trazodone, which resulted in uninterrupted sleep lasting 15 h. The patient was discharged with no medications and has remained asymptomatic on follow-up. The paranoia appears to be associated with the Peyote ingestion, while the visual/auditory hallucinations appear to be a result of insomnia.
Merlis 1957 [56]	Dose-ranging study	ROBINS-I/Serious	A total of 34 experiments with 24 patients, with 10 patients receiving two sessions of mescaline (0.5 g and 0.75 g of mescaline, respectively). Participants were questioned at intervals during four to six hours after injection with the mescaline. Questions included a psychodynamic profile. Seven patients experienced marked anxiety, apprehension, increased psychomotor activity, and intensification of hallucinations/delusions. A total of 17 patients developed tachycardia, facial flushing, and pupillary dilation, reporting they became conscious of inner feelings of tension/uneasiness but were otherwise emotionally stable. One participant out of the 24 (one of the aforementioned 7 patients with anxiety) had lasting reactions to mescaline. She appeared to be less disorganized than her baseline psychosis and was able to be discharged safely home shortly after without the need for rehospitalization. Five in the group of seven with anxiety and two in the group with feelings of tension were able to have partial improvements in their psychosis, but they all returned to their pre-mescaline state within four to six weeks.
Moreno 1997 [57]	Case report	JBI Case Report/Good	34-year-old male with a history of incapacitating OCD and prior polysubstance use. When the patient took psilocybin, his OCD symptoms improved for 4–5 days. Peyote cactus ingestion on five different occasions with similar improvements in OCD symptoms, but the psychedelic and adverse effects were more potent and longer lasting. Therefore, the patient began to use psilocybin daily for 4 years, but the patient developed a tolerance to it. After a few years of his symptoms returning to baseline, the patient began to take psilocybin again.
NCT04227756 2020 [58]	Double-blind, placebo-controlled, crossover design clinical trial	ROBINS-I/Low	Double-blind, placebo-controlled clinical trial of 32 healthy volunteers who found 500 mg of mescaline comparable to LSD/psilocybin, weaker at 300 mg. The Adjective Mood Rating Scale, 5 Dimensions of Altered States of Consciousness, the States of Consciousness Questionnaire, and visual analog scales were used to measure participant responses. Mescaline had a slower onset, longer duration, and induced more inactivity and subacute headaches.
Pennes 1954 [59]	Within-subjects study	ROBINS-I/Serious	Study of 55 schizophrenia patients documented experiences after taking mescaline, LSD, and amytal. Reported intensified symptoms of anxiety, paranoid manifestations, and thinking disorders.
Prince 2019 [60]	Cross-sectional survey study	NHLBI/Moderate	Cross-sectional study focusing on the relationship of American Indians and attributing their identity to the use of Peyote and religious purposes.
Rabinowitz 2023 [61]	Cross-sectional survey study	NHLBI/Moderate	Survey of 56,276 adults on the relationship between previous psychedelic use and prevalence of substance use disorder. Lifetime mescaline use was associated with lower odds of past-year substance dependence or abuse.
Reynolds 1985 [62]	Case report	JBI Case Report/Poor	Case report of a male who took mescaline, entered a hallucinogenic state, and engaged in the dangerous behavior where he “leaped” off a cliff.
Richards 1961 [63]	Single-arm trial	ROBINS-I/Moderate	Single-arm trial of 16 volunteers documenting repeated mescaline intoxication, illustrating distorted perceptions of environment and body, anxiety, and loss of control over their thoughts.
Rojas-Hernández 2024 [18]	Cross-sectional survey study	NHLBI/Moderate	Survey of 735 participants comparing Spanish vs. South American psychedelic use. Described mescaline use as a “deeply meaningful experience”.
Romeo 2025 [64]	Retrospective cohort study	NHLBI/Moderate	Survey of 1657 participants describing the intensity of psychedelic experiences based on which psychedelics were used.
Rosenberg 1986 [65]	Case series	NHLBI/Good	Case reports of 12 patients taking anticholinergics, one of whom later developed hyperthermia and severe muscle cramps. The goal is that hyperthermia should be looked out for.
Silva 1960 [66]	Within-subjects study	ROBINS-I/Serious	Study from 1960 of 4 subjects analyzing experiences of different psychotomimetics, but primarily of Taraxein. All 4 subjects received mescaline among other test psychotomimetics. One subject went into “rage”. Other reactions included anxiety, euphoria, and delusion.
Simonds 1980 [67]	Cross-sectional interview study	NHLBI/Moderate	Interviewing 109 juveniles with drug abuse in training school about their drug use, including mescaline. No significant correlation between mescaline use and violence or property offenses.
Simonsson 2023 [68]	Cross-sectional survey study	NHLBI/Moderate	Online survey of 953 adults that looked at potential effects of classic psychedelic usage with meditative practices. Compared those who did vs. did not use classic psychedelics across variables such as lifetime exposure to meditation, amount of meditation, percentage who feel glad to have practiced meditation/motivated to practice, perceived barriers/efficacy/knowledge to practicing. No increase in meditation-related adverse effects.
Simonsson 2023 [84]	Cross-sectional survey study	NHLBI/Good	Cross-sectional survey of 2822 adults from national database to draw insight into prevalence and association of challenges and adverse effects of psychedelic use to better understand methods for harm prevention/reduction. In total, 59.1% of respondents stated no distressing experience post-psychedelic use; 4.6% noted severely impaired functioning; and 8.9% reported impairment that lasted longer than 1 day. “Five most commonly reported helpful interventions during respondents’ most challenging, difficult, or distressing classic psychedelic experience were: trying to calm the mind, changing location, asking for help from friends, changing social environment, and smoking cannabis”.
Smith 1958 [69]	Comparative trial	ROBINS-I/Serious	Trial of 24 participants with adjuvant psychotherapy supplemented by occupational and recreational therapy, given a single dose of either 200–400 mcg LSD or 0.5 g of mescaline. Study noted that alcoholics appeared to be more resistant to the effects of LSD. No alcoholism was worsened due to treatment. Difficult to ascertain the contribution made by psychedelic drugs due to multiple treatment factors; however, study noted psychedelics as a promising adjunct to psychotherapy but not very useful as monotherapy. “Mescaline, on the whole, appeared to produce the more marked reactions” [compared to LSD].
Smith 2009 [70]	Case report	JBI Case Report/Good	Case report of 16 y/o Native American girl presenting to the ED with severe mescaline toxicity, found “convulsing and dry heaving” after drinking 12–16 oz of Peyote extract a few hours prior. “She received diazepam and naloxone before ET intubation and propofol. Routine lab studies, a UDS for drugs of abuse, head CT & LP were unremarkable but for high TSH.” Full recovery within 24 h. “1st time we have seen a case of MC toxicity confirmed by GC-MS in 26 years.”
Stone 2007 [71]	Cross-sectional survey study	NHLBI/Good	Survey of 114,241 participants found “mescaline use is associated with moderately strong excess risk of becoming hallucinogen dependent soon after onset”. “An estimated 2–3% of these recent-onset hallucinogen users had become dependent on hallucinogens”. “Very early first use of hallucinogens (age 10–11 years) is associated with increased risk of hallucinogen dependence (*p* < 0.01)”. “According to the estimates, both crude and covariate- adjusted, and with the LSD/NOS subgroup of users as a reference category, mescaline use is associated with moderately strong excess risk of becoming hallucinogen dependent soon after onset of hallucinogen use.”
Teitelbaum 1977 [72]	Case series	NHLBI/Good	Two cases. 1st case: 16-year-old male with history of known drug use, presented with 12 h of intense abdominal pain, cramping, and nausea, admitted for acute gastroenteritis and ruled out for acute surgical abdominal pain. Was prepared to have laparotomy performed, but later discovered to have used mescaline and was treated conservatively with fluids and antispasmodics. 2nd case: 18-year-old male with a long history of drug use found by family confused, disoriented, and hallucinating, with abdominal pain, nausea, and vomiting. Both patients thought they had ingested pills containing LSD, but later tested positive for mescaline through urine screening.
Thale 1950 [73]	Non-blinded clinical trial	ROBINS-I/Serious	Experimental study of 6 participants using mescaline to induce visual hallucinations, measured by color perception in an imagery test, then tested for mescaline excretion.
Uthaug 2022 [74]	Cross-sectional survey study	NHLBI/Good	Survey of 452 participants. Discusses benefits, side effects, and treatment profile. Psychological impairments of mescaline include: sensory alterations, ideas of influence, paranoia, delusions, and depersonalization. “Regarding abuse potential, most respondents (55%) indicated that they never consumed more than one dose of mescaline in a session, and approximately one-third (32%) reported that they have consumed mescaline with other substances on one or two occasions. Additionally, very few respondents reported craving mescaline (9%), ever being arrested or in legal trouble due to mescaline use (1%), or ever being in therapy or psychiatric treatment (<1%), and none reported seeking medical attention (0%) as a result of mescaline use.” “Most respondents with prior psychiatric conditions (i.e., depression, anxiety, post-traumatic stress disorder, and drug and alcohol misuse) reported improvements in these conditions following their most memorable experience with mescaline.” Results showed no significant differences in the subjective acute and enduring effects between types of mescaline.
Vina 2025 [75]	Cross-sectional survey study	NHLBI/Moderate	Survey of 458,372 adults found that psychedelic use was associated with levels of psychological distress.
Vina 2024 [76]	Cross-sectional survey study	NHLBI/Moderate	Cross-sectional survey study of 458,372 adults examining the association between psychedelic use and experienced stigma/formal treatment-seeking behaviors in US individuals. It was found that those with psychedelic use reported significantly higher levels of stigma, though only ayahuasca use was the only psychedelic not associated with stigma. Psychedelic users were more likely to use prescribed mental health medication. Mescaline use did not exhibit a statistically significant relationship with reduced likelihood of seeking treatment.
Wadsworth 1972 [77]	Case report	JBI Case Report/Poor	Case report of teenager who had been using mescaline for 2 years recreationally, finding it safe and pleasurable. Once starting college, she psychiatrically decompensated and told her psychiatrist that God was communicating to Earth through her and that “We are all here together and it is warm and nice.” She was described as delusional, prone to many errors, and required hospitalization.
Wolbach 1962 [78]	Single-blind crossover study	ROBINS-I/Serious	Study of 10 participants. Experiment 1 compared effects and determined equivalent doses of LSD, mescaline, and psilocin in 10 subjects. Experiment 2 examined the cross-tolerance between LSD and mescaline in 10 subjects. Of note, the participants in both experiments were the same; all had psychiatric diagnoses of character or personality disorders, and all had received LSD in previous experiments. Both LSD and mescaline resulted in increased body temperature, pulse rate, systolic blood pressure, and pupillary size. There was also a decreased threshold for elicitation of the knee jerk. LSD tartrate is about 2400 to 4900 times as potent as mescaline hydrochloride.
Yelmo-Cruz 2019 [79]	Case report	JBI Case Report/Poor	Case report of a 35-year-old male who was hospitalized for the second time due to psychotic symptoms with a positive drug screen for cannabis use and endorsement of consuming San Pedro cactus.
Zech 2025 [80]	Cross-sectional survey study	NHLBI/Good	Cross-sectional survey of 308,896 participants examining the association between psychedelics and cannabis use disorder. Past year use of mescaline had no significant relationship with past year cannabis abuse.
Zhu 2025 [81]	Case report	JBI Case Report/Moderate	Case report of 24-year-old Native American woman with a 3-week history of generalized weakness and paresthesia, which was preceded by two weeks of nausea, vomiting, and severe constipation. Guillain–Barré syndrome was suspected, but ruled out. Autoimmune encephalitis with polyneuropathy was also suspected and treated without improvement. It was discovered that her mother was regularly administering Peyote as part of a religious practice; it appears that Peyote may have led to the patient’s neurological symptoms.

**Table 2:** Prevalence of the psychological effects of mescaline.

Phenotype	Clinical Setting (G1)	Exposure Setting (G2)
Well-being	31.4% ± 6.1%	2.7% ± 5.2%
Anxiety/Agitation	18.8% ± 5.1%	32.4% ± 15.2%
Psychosis/Paranoia	4.8% ± 2.8%	67.6% ± 15.2%
No Change	45.0% ± 6.5%	2.7% ± 5.2%

**Table 3:** Prevalence of the somatic effects of mescaline.

Phenotype	Clinical Setting (G1)	Exposure Setting (G2)
Tachycardia	15.8% ± 4.6%	41.5% ± 14.9%
Mydriasis	6.7% ± 3.1%	24.4% ± 13.0%
Nausea/Vomiting	1.7% ± 1.6%	12.2% ± 9.9%
No Adverse	75.8% ± 5.4%	22.0% ± 12.5%

## Data Availability

No new data were created or analyzed in this study. Data sharing is not applicable to this article.

## Supplementary Materials

The following supporting information can be downloaded at: https://www.mdpi.com/article/10.3390/ijms27073081/s1.

## Abbreviations

Native American Church (NAC), Lysergic acid diethylamide (LSD).
