# Supplementary material for: The Ethnopharmacological Use of Mescaline for Psychiatric Disorders: A Systematic Review
Source: Int J Mol Sci. 2026 Mar 28;27(7):3081. doi: 10.3390/ijms27073081 (PMC13072975; doi:10.3390/ijms27073081)
Supplement: Supplementary file 1 [file ijms-27-03081-s001.zip › Supplementary File S1 PROSPERO Protocol.pdf]

PROSPERO International prospective register of systematic reviews

Review title and timescale

1 Review title
The Ethnopharmacological Use of Mescaline for Psychiatric Disorders: A Scoping Review

2 Original language title
English

3 Anticipated or actual start date
October 27, 2025

4 Anticipated completion date
December 31, 2025

5 Stage of review at time of this submission

Review stage	Started	Completed
Preliminary searches	x	x
Piloting of the study selection process	x	x
Formal screening of search results against eligibility criteria		
Data extraction		
Risk of bias (quality) assessment		
Data analysis		

Review team details

6 Named contact
Jonathan Shaw

7 Named contact email
jonathan.shaw@md.cusm.edu

8 Named contact address
1501 Violet St, Colton, CA 92324

9 Named contact phone number
N/A

10 Organizational affiliation of the review
None

11 Review team members and their organizational affiliations

Title	First name	Last name	Affiliation
Principle Investigator	Robert	Bota	Psychiatry, College Medical Center
Named Contact, co-investigator	Jonathan	Shaw	School of Medicine, California University of Science and Medicine
Co-investigator	Sagar	Pyreddy	School of Medicine, California University of Science and Medicine
Co-investigator	Jen-Yeu	Wang	School of Medicine, California University of Science and Medicine
Co-investigator	Emily	Ton	School of Medicine, California University of Science and Medicine
Co-investigator	Peter	Bota	School of Medicine, California University of Science and Medicine
Co-investigator	Anton	Andricioaei	Department of Anthropology, University of California, Riverside

12 Funding sources/sponsors
California University of Science and Medicine will provide support for this review by covering publication fees associated with publishing this review in a peer-reviewed academic journal. College Medical Center and University of California, Riverside will not provide any funding. The authors declare no sources of funding outside of the previously listed here.

13 Conflicts of interest
The authors declare no conflicts of interests

14 Collaborators
None

Review methods

15 Review question(s)
- What psychiatric conditions have mescaline-containing plants been used to treat as traditional medicine?
- How effective has mescaline been for the treatment of psychiatric disorders?
- What are the proposed mechanisms of action for mescaline?
- What is the safety profile of mescaline?

- What are the common sources of mescaline for recreational or self-medication purposes?
- What clinical trials and case reports are available detailing the clinical effects of mescaline?

16 Searches

- PubMed: ("Mescaline"[Mesh] OR “mescaline”[tiab] OR “peyote”[tiab] OR "Lophophora williamsii"[tiab] OR "Lophophora williamsii"[Mesh] OR "San Pedro cactus"[tiab] OR "Trichocereus pachanoi"[tiab] OR "Echinopsis pachanoi"[tiab]) AND ("Mental Disorders"[Mesh] OR "Psychiatry"[Mesh] OR "Psychiatric Disorder"[tiab] OR "Mental illness"[tiab] OR “Mental health”[tiab] OR "Mood disorder"[tiab] OR "Affective disorder"[tiab] OR “depression”[tiab] OR “depressive”[tiab] OR “anxiety”[tiab] OR "anxiety disorder"[tiab] OR "post-traumatic stress"[tiab] OR “PTSD”[tiab] OR "substance use"[tiab] OR “addiction”[tiab] OR "psychotic disorder"[tiab] OR “psychosis”[tiab] OR “schizophrenia”[tiab] OR “schizoaffective”[tiab] OR “bipolar”[tiab] OR "obsessive compulsive"[tiab] OR “OCD”[tiab] OR “suicidality”[tiab] OR “suicide”[tiab] OR "personality disorder"[tiab] OR "Ethnopharmacology"[Mesh] OR ethnopharmacology[tiab] OR “ethnobotany”[tiab] OR “traditional medicine”[tiab])
    - 433 references on 10/27/25
- Scopus: (TITLE-ABS-KEY) ("Mescaline" OR "mescaline" OR "peyote" OR "Lophophora williamsii" OR "San Pedro cactus" OR "Trichocereus pachanoi" OR "Echinopsis pachanoi") AND (TITLE-ABS-KEY) ("Mental Disorders" OR "Psychiatry" OR "Psychiatric Disorder" OR "Mental illness" OR "Mental health" OR "Mood disorder" OR "Affective disorder" OR "depression" OR "depressive" OR "anxiety" OR "anxiety disorder" OR "post-traumatic stress" OR "PTSD" OR "substance use" OR "addiction" OR "psychotic disorder" OR "psychosis" OR "schizophrenia" OR "schizoaffective" OR "bipolar" OR "obsessive compulsive" OR "OCD" OR "suicidality" OR "suicide" OR "personality disorder" OR "Ethnopharmacology" OR "ethnopharmacology" OR "ethnobotany" OR "traditional medicine")
    - 1,069 references on 10/27/25
- Embase: ('mescaline'/exp OR ‘mescaline’:ti,ab OR ‘peyote’:ti,ab OR 'lophophora williamsii':ti,ab OR 'lophophora williamsii'/exp OR 'san pedro cactus':ti,ab OR 'trichocereus pachanoi':ti,ab OR 'echinopsis pachanoi':ti,ab) AND ('mental disorder'/exp OR 'psychiatry'/exp OR 'psychiatric disorder':ti,ab OR 'mental illness':ti,ab OR 'mental health':ti,ab OR 'mood disorder':ti,ab OR 'affective disorder':ti,ab OR ‘depression’:ti,ab OR ‘depressive’:ti,ab OR ‘anxiety’:ti,ab OR 'anxiety disorder':ti,ab OR 'post-traumatic stress':ti,ab OR ‘ptsd’:ti,ab OR 'substance use':ti,ab OR ‘addiction’:ti,ab OR 'psychotic disorder':ti,ab OR ‘psychosis’:ti,ab OR ‘schizophrenia’:ti,ab OR ‘schizoaffective’:ti,ab OR ‘bipolar’:ti,ab OR 'obsessive compulsive':ti,ab OR ‘ocd’:ti,ab OR ‘suicidality’:ti,ab OR ‘suicide’:ti,ab OR 'personality disorder':ti,ab OR 'ethnopharmacology'/exp OR ‘ethnopharmacology’:ti,ab OR ‘ethnobotany’:ti,ab OR 'traditional medicine':ti,ab)
    - 1,247 references on 10/27/25
- Cochrane: ("mescaline" OR mescaline:ti,ab,kw OR peyote:ti,ab,kw OR "lophophora williamsii":ti,ab,kw OR "lophophora williamsii" OR "san pedro cactus":ti,ab,kw OR "trichocereus pachanoi":ti,ab,kw OR "echinopsis pachanoi":ti,ab,kw) AND ("mental disorder" OR "psychiatry" OR "psychiatric disorder":ti,ab,kw OR "mental illness":ti,ab,kw OR "mental health":ti,ab,kw OR "mood disorder":ti,ab,kw OR "affective disorder":ti,ab,kw OR depression:ti,ab,kw OR depressive:ti,ab,kw OR anxiety:ti,ab,kw OR "anxiety disorder":ti,ab,kw OR "post-traumatic stress":ti,ab,kw OR ptsd:ti,ab,kw OR "substance use":ti,ab,kw OR addiction:ti,ab,kw OR "psychotic disorder":ti,ab,kw OR psychosis:ti,ab,kw OR schizophrenia:ti,ab,kw OR schizoaffective:ti,ab,kw OR bipolar:ti,ab,kw OR "obsessive compulsive":ti,ab,kw OR ocd:ti,ab,kw OR suicidality:ti,ab,kw OR suicide:ti,ab,kw OR "personality disorder":ti,ab,kw OR "ethnopharmacology" OR ethnopharmacology:ti,ab,kw OR ethnobotany:ti,ab,kw OR "traditional medicine":ti,ab,kw)
    - 20 results (3 reviews, 1 protocol, 16 trials) on 10/27/25

17 URL to search strategy

N/A

18 Condition or domain being studied

This scoping review examines the use of mescaline-containing plants for the treatment of psychiatric disorders, either as a form of self-medication in line with traditional medicine practices, as a recreational substance, or as an intervention used as part of a clinical trial. All psychiatric disorders will be considered in this scoping review.

PICOST: Mescaline, Traditional, complementary, and alternative medicine, Mental Health Treatment

19 Participants/population

Inclusion criteria: Individuals of any age who have used mescaline, particularly through “Peyote” (Lophophora williamsii) or the “San Pedro cactus” (Trichocereus pachanoi). References and studies containing original patient data or the opinions/beliefs of participants will be included.
Exclusion criteria: Individuals who have not used mescaline will be excluded. Additionally, references which do not include original patient data or is otherwise not a primary source regarding the opinions/beliefs of the use of mescaline-containing plans as traditional medicine will be excluded.

20 Intervention(s), exposure(s)

Inclusion criteria for interventions: Mescaline or mescaline-containing sources like “Peyote” (Lophophora williamsii) or the “San Pedro cactus” (Trichocereus pachanoi)
Exclusion criteria for interventions: None

21 Comparator(s)/control

None

22 Types of study to be included

Give details of the study designs to be included in the review. If there are no restrictions on the types of study design eligible for inclusion, this should be stated.

Included:

- Studies with original patient data (case reports, case series, randomized and non-randomized controlled trials)
- Studies that are primary sources for beliefs/opinions on the use of mescaline-containing plants as traditional medicine
- English full-text must be available

Excluded:
- Unpublished studies (trial protocols)
- Animal studies

23 Context
Studies in any healthcare setting in any country/geographical region with no timeframe limits for publication.

24 Primary outcome(s)
- Efficacy of mescaline or mescaline-containing plants for the treatment of psychiatric disorders

25 Secondary outcomes
- Safety/side effect profile of mescaline use
- Quality of reporting for patient data and outcomes

26 Data extraction (selection and coding)
Studies will be selected by two authors independently by screening titles and abstracts in (Covidence systematic review management program). The selected studies will be subject to full text screening by applying the selection criteria. Reasons for exclusion will be documented in the program. Any discrepancies between authors will be adjudicated by a third author. Two authors will independently extract the following data: study design, study population, and outcome measures. Where possible, missing values (e.g. standard deviation) will be calculated from the available data (p-values, t-values, confidence intervals or standard errors). Authors/investigators may be contacted regarding missing data or if results of clinical trials were no publicly released.

27 Risk of bias (quality) assessment
The GRADE approach will be used to determine the certainty in evidence for each outcome deemed critical or important. GRADE assesses the limitations in study design, indirectness, imprecision, inconsistency and publication bias. For experimental studies the limitations in study design of the individual studies will be determined by checking if there is lack of allocation concealment, lack of blinding, incomplete accounting of outcome events, selective outcome reporting and/or other limitations. The relevant risk of bias instruments (Cochrane risk of bias tool, Joanna Briggs Institute Critical Appraisal Checklist for Case Reports, SANRA, and NHLBI Study Quality Assessment Tool for Case Series) will be used.

28 Strategy for data synthesis
We will use IBM SPSS Statistics 28.0.1.0 for data analysis. Data synthesis will be conducted using the Covidence systematic review management program using a standardized data extraction template. This template will include the name of the primary author, country of publication, year of publication, study type, number of patients, primary and secondary outcomes measured (as described elsewhere in the protocol), and an optional free response section for notes for unique aspects of a study for the included references. Quantitative synthesis is planned, and a meta-analysis will be performed with evidence level 4 and above. Qualitative analysis of themes in interviews will also be performed.

We will report continuous outcomes as standardized mean differences (SMD) with 95% Cis using different scales present in the included references. Dichotomous outcomes will be reported as Risk Ratios (RR) with 95% CIs. Heterogeneity will be assessed by visual inspection of the forest plot, by using the Chi²-test (significant if $p < 0.10$) and the I^2 statistic (heterogeneity considered significant if $I^2 > 60\%$). In case of heterogeneity, meta-analysis might not be carried out.
If two or more studies of similar design on the same intervention and assessing the same outcome and sufficient data are available, meta-analyses will be performed. Since we anticipate variation between studies, meta-analysis will be carried out using the random effects model. The Mantel-Haenszel method will be used for dichotomous outcomes, and the Inverse Variance method will be used for continuous outcomes. A p-value < 0.05 will be considered significant.

29 Analysis of subgroups or subsets
The following subgroup analyses could be performed, if sufficient data is available:
- patient gender
- patient age group
- patient race/ethnic background
- psychiatric disorder treated

Review general information

30 Type and method of review
Scoping Review and Meta-analysis (if done)

31 Language
This review will be completed fully in English

32 Country
United States of America

33 Other registration details
N/A

34 Reference and/or URL for published protocol
N/A

35 Dissemination plans
The authors intend to publish the review on completion

36 Keywords
Mescaline, Traditional, complementary, and alternative medicine, Mental Health Treatment, Peyote, San Pedro Cactus

37 Details of any existing review of the same topic by the same authors
None

38 Current review status
On-going

39 Any additional information
None

40 Details of final report/publication(s)
This field will be filled in once this review has been published
